# Prevalence of psychiatric symptoms in children and adolescents one year after the 2009 L’Aquila earthquake

**DOI:** 10.1186/s12888-014-0270-3

**Published:** 2014-09-24

**Authors:** Paolo Feo, Simona Di Gioia, Emanuela Carloni, Benedetto Vitiello, Alberto Eugenio Tozzi, Stefano Vicari

**Affiliations:** Department of Neuroscience, Child and Adolescent Neuropsychiatry Unit, Bambino Gesù Children’s Hospital, IRCCS, Piazza Sant’Onofrio 4, 00165 Rome, Italy; Multifactorial Diseases and Complex Phenotypes Research Area, Bambino Gesù Children’s Hospital, IRCCS, Rome, Italy; National Institute of Mental Health, Bethesda, Maryland USA

**Keywords:** Abruzzo, Child and adolescent, Mental health, CBCL, Post traumatic stress disorder

## Abstract

**Background:**

In 2009, an earthquake devastated the Abruzzo region in Italy. Despite the occurrence of several disasters in this country, no study on mental health of Italian children has ever been conducted in complex emergencies. Objective of the study was to assess the prevalence of psychiatric symptoms among children in the affected area 12 to 17 months after the event.

**Methods:**

A community sample of 1839 3-14 years children was identified from the general population assigned to 37 paediatricians of the National Health System, including children living in the earthquake epicentre, the surrounding earthquake zone, and the adjacent non-affected areas. Psychiatric symptoms were assessed with the Child Behavior Checklist (CBCL) and the Youth Self Report (YSR), completed by 452 children aged 11–14 years. The association between symptoms and sociodemographic, health, family, and earthquake-related factors was examined.

**Results:**

The prevalence of CBCL-defined cases was 14.9% in the epicentre, 13.0% in the remainder earthquake zone, 13.9% in the unaffected area (p = .876). No differences among areas were found when comparing the YSR results. Prevalence of CBCL-defined post-traumatic stress (PTS) cases was 8.4% in the epicentre, 4.0% in the remainder earthquake zone, 2.2% in the unaffected area (p = .002). PTS and anxiety were significantly more frequent in the epicentre than in other areas only in the 6–10 year-old children group (respectively p = .009 and p = .014). In multivariate logistic analyses, factors associated with PTS were living in the epicentre (OR = 3.6) and child or maternal history of mental health care prior to the earthquake (respectively OR = 7.1 and OR = 4.5).

**Conclusions:**

Children living in the epicentre, particularly those 6–10 years old, had the highest prevalence of CBCL-defined cases, and of PTS and anxiety symptoms one year after the earthquake. No signs of increased psychopathology were detected in younger (3–5 years) or older children (11–14 years). Family and health related factors showed stronger association with psychiatric outcomes than earthquake-related factors. The identification of populations at higher risk of developing psychiatric symptoms has implications for public health interventions in complex emergencies.

## Background

On April 6, 2009 an earthquake measuring 5.9 on the Richter scale hit the city of L’Aquila and the surrounding area in the Abruzzo Region, Italy. The event affected 57 municipalities, killed 308 persons, injured more than 1,600 people and displaced more than 67,000 people. It wreaked havoc with Abruzzo infrastructures, historic and artistic patrimonies [1], causing over ten million euro in damages, according to the official estimates by the Italian Government. As of September 2012, more than 32,000 people are still displaced, benefiting from various governmental housing programmes [2].

Exposure to potentially traumatic events may be associated with a wide range of psychiatric disorders in children and adolescents. These conditions can have variable degrees of comorbidity, and a long-lasting impact on quality of life, and academic achievements [3-9].

Knowledge of the consequences of natural disasters on child and adolescent mental health is limited for several reasons. First, most studies conducted in the aftermath of disasters focused on post-traumatic stress (PTS) symptoms or PTS disorder (PTSD) and depressive symptoms or disturbances only. Secondly, most tools used to assess the mental health outcomes present unsatisfactory levels of sensitivity and specificity [6,10-12].

A meta-analysis of PTS in children after natural disasters [11] shows an association between the event and PTS (d = 0.4), but the same meta-analysis and a systematic review of psychopathology in children survivors of disasters [12] also highlight a great variability in the disorders prevalence. In fact, in example, with regard to children exposed to earthquakes, studies show a PTSD prevalence of 2.5-95% [13,14] and a depression prevalence of 5.4-76% [14,15].

Focusing on data more relevant to the present study, between 12 and 18 months after natural disasters Wang et al. [12] report that data from preschool children community samples are lacking, while for school age children and early adolescents self-reported prevalence of PTS and depression symptoms ranges from, respectively, 11.2% and 11.3% at 12 months [16], to 12.4% and 13.9% at 15 months [17], to 95% and 76% at 18 months after an earthquake [14]. Prevalence tend to be lower when data are collected through a psychiatric interview: according to DSM-IV criteria [18], PTSD prevalence is found 2.1%-4.7% 18 months after floods [19,20], while depression prevalence is found 5.4% 18–20 months after an earthquake [15]. Nevertheless this trend is not absolute: when investigating very highly affected populations, even studies based on diagnostic assessment according to ICD-10 [21] found PTSD and depression prevalence of 30.6% and 23.7%, respectively, 12 months after a super-cyclone [22], and others according to DSM-IV found 31.6% PTSD and 5.5% depression 12 months, and 10.4% PTSD 18 months after the 2004 tsunami [23,24]. One of these two studies also examined preschool children; in the whole sample, thus including tsunami affected and non affected children, at this age prevalence of psychiatric disorders was found lower than in school age children (2.3% vs 3.8-16.6%) 1 year after the tsunami [23].

Methodological proposed explanations for the prevalence variability include differences of studied populations, time of assessment since the disaster and the use of self-report instruments [6,10,11,25].

To reduce biases generated by single informants, a multi-informant assessment in post-disaster settings may be adopted. However, to our knowledge, this model was applied only to refugee children with parents reporting a higher prevalence of psychiatric symptoms than children; however, there was no clear cut explanation for this finding [26].

Severity of exposure to disasters, i.e. magnitude of the event, proximity to the epicentre, losses, perceived threat, are the most evident risk factors for children mental health. Female gender is often associated with PTS and PTSD. The role played by general mental health and reaction to trauma of parents and by the child’s age on PTS symptoms is still a matter of debate [11]. Moreover, the frequent use of bivariate analysis in associating exposures with outcomes may miss the effect of potential confounders and other determinants of outcomes that would be best estimated by multivariate analysis [27,28].

Despite the disasters that have occurred in Italy during the last decades, only a few studies focused on children and adolescents mental health. Six months after the 2002 earthquake in Molise, PTSD prevalence was 14.5% in an over 14 years community sample; however, data on adolescents were not disaggregated from those on adults [29].

Prevalence of PTSD in students attending the last year of high school in L’Aquila was 37.5% ten months after the earthquake and 30.7% twenty-one months after [30,31]. Additionally, 66.7% people aged seventeen to thirty years seeking help at the L'Aquila University Psychiatry Unit self-reported the presence of PTS symptoms in the first nine months after the earthquake; 13.8% were diagnosed with PTSD according to DSM-IV [18,32]. Children and adolescents affected by autism spectrum disorder who had experienced the L’Aquila earthquake showed a decline in adaptive behaviour at six months and at one year after the disaster [33]. Despite their public health relevance, no data are available on the prevalence of psychiatric symptoms in children and adolescents out of the general population after the L’Aquila earthquake.

Primary aims of the present study were to (i) measure the prevalence of a broad range of psychiatric symptoms in a community sample of children and pre-adolescents in Abruzzo after the 2009 earthquake, evaluating differences in outcomes between children resident in zones hit by the earthquake and those living in surrounding non-affected areas, and (ii) investigate the association of sociodemographic, health, family, and earthquake-related factors with the psychiatric outcomes.

### Setting

L’Aquila is the capital of Abruzzo in Central Italy. Abruzzo’s population is over 1.3 million; the population under 18 years of age is over 210 thousand [34].

The National Health System provides full care coverage at no out–of-pocket cost through a network of general practitioners and family paediatricians (FPs). They maintain clinical records of all the residents and are connected with a capillary network of National Health System multidisciplinary community health units.

Ministry of Education ensures homogeneity of education system that is freely accessible and education is compulsory up to the age of 16.

## Methods

### Study design and sample

This cohort study included the follow up of a group of children exposed to the earthquake. The first phase, which has a cross sectional design, aimed at assessing the prevalence of psychopathological symptoms in a community sample of children aged 3–14 years by using screening instruments. The second phase aimed at ascertaining the presence of psychiatric disorders according to DSM-IV in those cases screened in the first phase by a semi-structured clinical interview. This survey reports results of the first phase.

**Figure 1:**
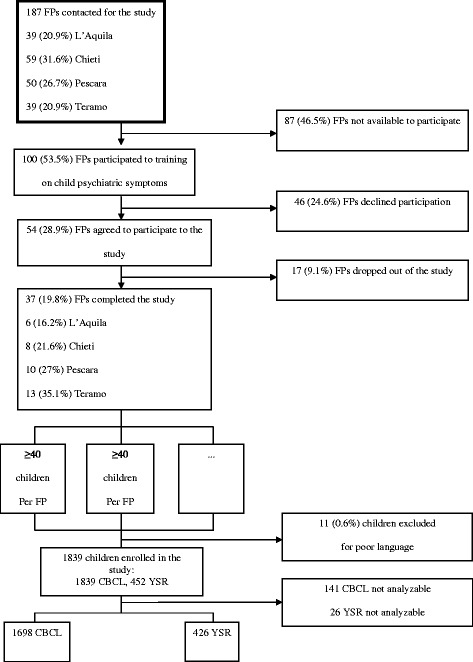
**Flow diagram of the study selection process and study subsets.**

Psychopathological symptoms were screened 12 to 17 months after the disaster in a convenience sample. We contacted all Abruzzo FPs (n = 187). One hundred FPs agreed to participate and attended a preliminary training on clinical management of traumatic reactions in children, family counselling and research methodology. Fifty-four out of these FPs initially accepted to participate to the study, but after 17 dropped out, eventually, 37 FPs completed the research. The proportion of the 37 participating FPs did not significantly differ among provinces (χ^2^_3_ = 6.4, p = 0.093) (Figure 1).

All children and adolescents aged 3 to 14 years assigned to the participating FPs were eligible for participation, while families with insufficient knowledge of the Italian language were excluded.

To estimate the prevalence of psychiatric symptoms with a precision of ±2%, assuming an expected prevalence of 20%, a power of 80% and a confidence level of 95%, a sample size of 1448 subjects, over a reference population of 25,000, was calculated.

Each participating FP enrolled at least 40 children in sequential order of presentation to consultation, in order to reach a total sample of at least 1448 children. Given the full coverage of the FPs network, each enrolling reservoir was assumed as corresponding to the total population of children and adolescents of that age resident in the same area of the assigned FP. The total enrolled sample consisted of 1850 children. Eleven children were excluded for their parents’ insufficient knowledge of the Italian language (Figure 1).

A total of 1839 children aged 3–14 years (mean 8.3 years, SD 3.2) from all parts of Abruzzo participated in the screening. As mentioned above, their residence was assumed as corresponding to that of the assigned FP: 160 were resident in L’Aquila, the earthquake epicentre, 215 in the remainder earthquake zone, 1464 in the rest of the Region. Fifty-five children were conservatively considered as living in the unaffected area, though their assigned 3 FP were resident in the same area at the border with the earthquake zone. All children were screened using the Child Behavior Checklist (CBCL) [35,36] and 452 children aged 11–14 were assessed by the Youth Self Report (YSR) [37]; 367 children aged 11–14 were evaluated by both tools. One hundred forty-one CBCL and 26 YSR were excluded due to incomplete data, so that valid CBCL were completed for 1698 children (92.3% of the targeted sample) and valid YSR for 426 children (94.3%) (Figure 1). All children participating in the study were regularly attending the school. The vast majority of children were Caucasian and only 55 were from different ethnic groups.

The study was approved by both the Ethical Committee of Bambino Gesù Children's Hospital IRCCS and of the local National Health System health units. Informed written consent was obtained by the parents.

### Measures

#### Sociodemographic, medical health, family and earthquake-related factors

An ad hoc questionnaire was developed and filled in by both parents, separately. Data concerning children were collected from their mothers. Sociodemographic, health and family data were: gender, age, parents’ level of education, mother’s marital status, current chronic physical disease, lifetime neuro-psychiatric condition, child’s and parents mental health care sought or received after and before the earthquake, distinctly. Earthquake-related factors were: proximity to the epicentre (resident in the earthquake epicentre, remainder earthquake zone, unaffected areas), damages to the house, (past or present) internally displaced condition, parents’ loss of job. When examining the association of these factors to CBCL outcomes, gender was analyzed as female versus male; age classes as, distinctly: age 6–10 years and 11–14 years versus 3–5 years; parents’ education as at least a high school diploma versus not holding it; mother’s marital status as married versus other conditions; child’s proximity to disaster as, distinctly: resident in L’Aquila and in the remainder earthquake zone versus resident in unaffected areas; damages to the house as uninhabitable versus inhabitable.

#### Psychiatric symptoms

A multi-informant assessment approach was adopted using the CBCL and the YSR (self-report questionnaire of the CBCL package for children aged over 10 years). Above instruments investigate psychopathological symptoms in the Italian population [38], and were previously used in complex emergencies [26,39-43].

The children’s psychiatric symptoms were measured by administering to parents the Child Behavior Checklist 1.5-5 or the Child Behavior Checklist 6–18 [36], according to the children’s respective ages. Children aged 11–14 also filled the Youth Self Report (YSR) [25]. The Italian versions of CBCL and YSR were obtained through back-translation authorized and approved by T. Achenbach [38]. Cut-off points of clinical interest were identified according to the tool kit software standards: norms were based on 90^th^ percentile (Tscore = 63) for Internalizing, Externalizing and Total Problems Scales, and on 97^th^ percentile (Tscore = 69) for DSM-IV oriented and PTS scales [44]. Children were identified as subjects of public health interest, hereafter referred to as cases, when rated as clinical at Internalizing, or Externalizing, or Total Problems Scales. Main Symptoms patterns were identified in Anxiety and Affective DSM-related Scales and PTS Scale.

### Data analysis

CBCL- and YSR-defined cases and symptoms prevalence was examined by being resident in L’Aquila, in the remainder earthquake zone, or in the unaffected areas. Chi-square was used to compare proportions. Step-wise forward multivariate logistic regression analysis was used to evaluate the effect of sociodemographic, health, family and earthquake-related factors on CBCL-defined cases and Main Symptoms that showed statistically significantly differences among residence groups in the whole sample. Odds ratios (ORs) were calculated to indicate the strength of association. Statistical significance was estimated using two-sided .05 tests. The independent variables included in the multivariate regression model had showed at least .20 statistical significance at univariate logistic regression analysis. 95% confidence intervals (CI) were calculated for symptoms prevalence and odds ratio values.

## Results

### Sociodemographic, health, family and earthquake-related factors

The sociodemographic characteristics of the sample are summarized in Table 1. Most parents had at least a high school education, with mothers more frequently than fathers (77.4% vs 66%). While no statistically significant difference was observed among fathers from different residence groups, this was observed among mothers (91.0% in L’Aquila vs 67.0% in the remainder earthquake zone vs 74.8% in unaffected areas, χ^2^_2_ = 28.0, p < .001). Prevalence of children’s chronic physical diseases and neuropsychiatric conditions was relatively low (respectively 3.3% and 2.4%). Mental health care seeking for children was 5.1% before the earthquake, and 2.9% after the earthquake. In this case, L’Aquila children show a statistically significant higher prevalence than other residence groups (9.5% vs 3.3% vs 2.0%, χ^2^_2_ = 28.4, p < .001).

**Table 1:** **Sociodemographic, medical health, family and earthquake-related factors of children by proximity to disaster**

		**Epicentre**		**Remainder earthquake zone**		**Unaffected areas**		**Total**		**p**
		**N**	**%**	**N**	**%**	**N**	**%**	**N**	**%**	
Gender	Females	80	50.0	106	49.5	743	51.0	929	50.7	-
	Males	80	50.0	108	50.5	714	49.0	902	49.3	-
Age	3-5 yrs	48	30.0	63	29.3	467	31.9	578	31.5	-
	6-10 yrs	73	45.6	98	45.6	608	41.6	779	42.4	-
	11-14 yrs	39	24.4	54	25.1	388	26.5	481	26.2	-
Parents’ high school diploma	Mother (N = 1703)	141	91.0	132	67.0	1011	74.8	1284	75.4	0.000
	Father (N = 1611)	109	73.2	122	62.6	832	65.7	1063	66.0	0.106
Mother not married (N = 1781)		7	4.4	18	8.5	127	9.0	152	8.5	0.148
Chronic physical diseases (N = 1766)		8	5.0	4	1.9	47	3.4	59	3.3	0.255
Lifetime Neuropsychiatric condition (N = 1678)		8	5.4	8	3.9	25	1.9	41	2.4	0.011
Pre-earthquake mental health care (N = 1755)		10	6.3	13	6.1	67	4.8	90	5.1	0.563
Post-earthquake mental health care (N = 1745)		15	9.5	7	3.3	28	2.0	50	2.9	0.000
Parents’ pre-earthquake mental health care	Mother (N = 1749)	13	8.2	9	4.2	54	3.9	76	4.4	0.045
	Father (N = 1671)	3	1.9	8	3.9	20	1.5	31	1.9	0.066
Parents’ post-earthquake mental health care	Mother (N = 1735)	10	6.3	4	1.9	19	1.4	33	1.9	0.000
	Father (N = 1655)	4	2.6	3	1.5	12	0.9	19	1.2	0.175
Present in the earthquake zone during the disaster (N = 1745)		152	98.7	172	82.7	63	4.6	387	22.2	0.000
Uninhabitable home after the earthquake (N = 1586)		117	74.1	23	10.9	176	14.5	316	19.9	0.000
Internally displaced (N = 1464)		153	96.2	41	20.0	50	4.6	244	16.7	0.000
Parents lost their job after the earthquake	Mother (N = 1835)	22	13.8	4	1.9	8	0.6	34	1.9	0.000
	Father (N = 1835)	19	11.9	0	0.0	10	0.7	29	1.6	0.000

*Note*: The number of observations varies across the items because of missing values.

Differences among the subsamples from different areas are calculated by χ^2^ test.

More mothers than fathers had sought mental health care both before and after the earthquake, mothers in the epicentre had higher mental health services utilization than mothers in other areas both before the earthquake (8.2% vs 4.2% vs 3.9%, χ^2^_2_ = 6.2, p = .045) and afterwards (6.3% vs 1.9% vs 1.4%, χ^2^_2_ = 18.3, p < .001). Fathers in the epicentre showed increased mental health services utilization after the earthquake from 1.9% to 2.6%.

A total of 22.2% of the children enrolled in the study were in the earthquake zone at the moment of the disaster. All earthquake-related factors show a statistically significant higher prevalence in L’Aquila (p < .001 for all factors). Here uninhabitable houses were 74.1%, internally displaced children 96.2%, and parents’ loss of job was 13.8% for mothers and 11.9% for fathers (Table 1).

### Psychiatric symptoms

Prevalence of total CBCL-defined cases was 13.9% (14.9% in L’Aquila, 13.0% in the remainder earthquake zone, 13.9% in unaffected areas); their distribution through age groups was significantly different (10.8% at 3–5 years, 15.9% at 6–10 years, and 14.3% at 11–14 years, χ^2^_2_ = 6.9, p = .032); the highest prevalence of CBCL-defined cases was found in children aged 6–10 in L’Aquila (22.9%). Prevalence of total YSR-defined cases was 4.5% (7.9% in L’Aquila, no cases in the remainder earthquake zone, 4.7% in unaffected areas) (Table 2).

**Table 2:** **CBCL and YSR-defined Cases by age groups and proximity to disaster and symptoms at CBCL by proximity to disaster**

	**Epicentre**	**Remainder earthquake zone**	**Unaffected areas**	**Total**		**p**
	**(%)**	**(%)**	**(%)**	**N**	**%**	
**CBCL-defined cases**						
Total sample	14.9	13.0	13.9	1698	13.9	0.876
Children 3-5ys	8.5	10.3	11.1	545	10.8	0.853
Children 6-10ys	22.9	13.4	15.5	741	15.9	0.218
Children 11-14ys	8.1	15.4	14.9	412	14.3	0.525
**YSR-defined cases**						
Children 11-14ys	7.9	0.0	4.7	426	4.5	0.198
**CBCL symptoms**						
Total	9.2	6.5	6.9	1698	7.1	0.465
Internalizing	10.6	13.6	11.7	1698	11.7	0.674
Externalizing	5.3	5.8	4.0	1698	4.4	0.450
Affective	5.2	2.9	3.3	1698	3.4	0.426
Anxiety	11.0	5.3	6.7	1698	6.9	0.085
PTS^a^	8.4	4.0	2.2	1153	3.0	0.002
Somatic^a^	5.6	0.7	2.8	1151	2.8	0.060
ADH	2.4	3.3	2.2	1698	2.4	0.738
Oppositional defiant	1.0	1.3	0.5	1698	0.6	0.436
Conduct^a^	0.7	0.9	0.8	1153	0.8	0.972
Pervasive development^a^	1.7	0.0	1.6	545	1.5	0.680

*Note*: Valid numbers (N) are expressed as quantities, all other data are expressed in percentages (%).

Differences among the subsamples from different areas are calculated by χ^2^ test.

ADH, attention deficit and hyperactivity; CBCL, Child Behavior Checklist; PTS, post-traumatic stress; YSR, Youth Self Report.

^a^PTS, Somatic and Conduct Scales are available only in the CBCL 6–18 form; Pervasive Development Scale is available only in the CBCL 3–5 form.

Internalizing symptoms were more frequent than externalizing symptoms either in the total sample (11.7% vs 4.4%) and in each residence group. Main Symptoms were always more frequent in L’Aquila, but only PTS showed statistically significant differences among residence groups (8.4% vs 4.0% vs 2.2%, χ^2^_2_ = 13.0, p = .002) (Table 2).

Analysis of the prevalence of CBCL and YSR symptoms in the three residence groups was conducted separately for the three age ranges. The only statistically significant differences were found in the 6–10 years group, concerning Main Symptoms: anxiety (15.7% vs 6.2% vs 6.3%, χ^2^_2_ = 8.5, p = .014) and PTS (10.0% vs 4.1% vs 2.8%, χ^2^_2_ = 9.3, p = .009). Affective problems showed the same prevalence pattern but did not achieve statistical significance (10.0% vs 5.2% vs 3.8%, χ^2^_2_ = 5.5, p = .064) (Table 3).

**Table 3:** **Prevalence of CBCL- and YSR-defined cases and symptoms at the CBCL and YSR scales by age group and proximity to disaster**

	**CBCL 3–5 ys**					**CBCL 6–10 ys**					**CBCL 11–14 ys**					**YSR 11–14 ys**				
	**Earthquake epicentre**	**Remainder earthquake zone**	**Unaffected areas**	**Total**	**p**	**Earthquake epicentre**	**Remainder earthquake zone**	**Unaffected areas**	**Total**	**p**	**Earthquake epicentre**	**Remainder earthquake zone**	**Unaffected Areas**	**Total**	**p**	**Earthquake epicentre**	**Remainder earthquake zone**	**Unaffected areas**	**Total**	**p**
Valid Number	47	58	440	545		70	97	574	741		37	52	323	412		38	46	342	426	
**Cases**	8.5	10.3	11.1	10.8	0.853	22.9	13.4	15.5	15.9	0.218	8.1	15.4	14.9	14.3	0.525	7.9	0.0	4.7	4.5	0.198
***Problems***																				
Total	2.1	10.3	6.1	6.2	0.219	10.0	8.3	7.1	7.6	0.669	5.4	9.6	7.4	7.5	0.752	7.9	0.0	2.9	3.1	0.106
Internalizing	6.4	6.9	9.8	9.2	0.610	21.4	11.3	12.9	13.5	0.114	8.1	13.5	12.1	11.9	0.727	7.9	0.0	3.2	3.3	0.128
Externalizing	2.1	5.2	2.7	2.9	0.551	8.6	6.2	5.1	5.5	0.456	5.4	3.9	4.0	4.1	0.918	0.0	0.0	1.8	1.4	0.474
Affective	0.0	0.0	3.2	2.6	0.180	10.0	5.2	3.8	4.6	0.064	2.7	1.9	2.5	2.4	0.965	0.0	0.0	0.9	0.7	0.690
Anxiety	6.4	1.7	7.3	6.6	0.278	15.7	6.2	6.3	7.2	0.014	8.1	7.7	6.8	7.0	0.940	2.6	0.0	1.2	1.2	0.537
PTS	-	-	-	-	-	10.0	4.1	2.8	3.6	0.009	5.4	3.9	1.2	1.9	0.125	2.6	0.0	0.9	0.9	0.445
Somatic	-	-	-	-	-	2.9	0.0	1.9	1.8	0.314	10.8	1.9	4.3	4.6	0.126	2.6	0.0	2.3	2.1	0.569
ADH	4.3	3.5	2.1	2.4	0.547	4.3	1.0	2.6	2.6	0.417	0.0	3.9	1.9	1.9	0.420	2.6	2.2	1.5	1.6	0.827
Oppositional defiant	0.0	1.7	0.5	0.6	0.408	2.9	1.0	0.9	1.1	0.316	0.0	0.0	0.0	0.0	-	2.6	0.0	1.5	1.4	0.585
Conduct	-	-	-	-	-	0.0	1.0	0.5	0.5	0.664	2.7	0.0	1.2	1.2	0.516	0.0	0.0	0.9	0.7	0.690
Pervasive development	0.0	1.7	1.6	1.5	0.680	-	-	-	-	-	-	-	-	-	-	-	-	-	-	-

*Note:* Valid numbers are expressed as quantities, all other data are expressed in percentages.

Differences among the subsamples from different areas are calculated by χ^2^ test.

ADH, attention deficit and hyperactivity; CBCL, Child Behavior Checklist; PTS, post-traumatic stress; YSR, Youth Self Report.

### Association of sociodemographic, health, family and earthquake-related factors with CBCL-defined cases and main symptoms that showed significantly different distributions

CBCL-defined cases were equally strongly predicted by child’s pre-earthquake and mother’s post-earthquake mental health care seeking (respectively: OR = 5.7, 95% CI = 3.5-9.4, p < .001; OR = 5.6, 95% CI = 2.7-11.7, p < .001). Child’s chronic diseases were also associated to CBCL-defined cases (OR = 2.0, 95% CI = 1.0-4.0, p = .047) even if with lower intensity and statistical significance, while father’s education showed to be a protective factor (OR = 0.7, 95% CI = 0.5-1.0, p = .041).

Both PTS and anxiety symptoms, the first more than the second, were predicted by child’s and mother’s pre-earthquake mental health care seeking (child: OR = 7.1, 95% CI = 3.1-16.2, p < .001 for PTS; OR = 4.0, 95% CI = 2.2-7.2, p < .001 for anxiety; mother: OR = 4.5, 95% CI = 1.7-11.8, p = .002 for PTS; OR = 3.2, 95% CI = 1.7-6.1, p < .001 for anxiety). PTS was the only condition predicted by earthquake direct related factors as living in L’Aquila, the epicentre (OR = 3.6, 95% CI = 1.5-8.6, p = .004).

## Discussion

To the best of our knowledge, this is the first study investigating the prevalence of psychiatric symptoms in an Italian community sample of children and adolescents after a natural disaster.

Children who needed psychiatric care 12 to 17 months after the earthquake were about 14% of the examined population. This proportion is consistent with that reported by parents (about 15%) in a representative sample of children and adolescents 2 years after the Hurricane Katrina [45].

The prevalence of internalizing symptoms (about 12%) was found equal to that found through diagnostic interview in a large random community sample of children and adolescents 18 months after the Hurricane Georges [46]. Within internalizing spectrum the expression of anxiety symptoms was higher than that of PTS and affective symptoms across age and proximity to disaster; an analogous pattern was observed in school age children and early adolescents after the Sichuan earthquake [16,47], and in adolescents after the Hurricane Georges [48].

PTS symptoms increased in the whole epicentre community likewise found elsewhere 15 months after an earthquake [16,17], but the rise was more pronounced in the children aged 6–10. In this group, prevalence of risk of psychiatric disorders (22.9%) reached levels predicted by the World Health Organization during the first year after disaster (23-24%) [49]. This finding would support that such estimated prevalence may remain stable or only gradually decrease over the first year in children and adolescents, or at least in specific groups, as documented by other studies [15,22-24,45,50-52].

The comparison with previous investigations in Italian population, although limited by the substantial lack of data, allows to evidence one closely methodological interesting point. We reported a higher prevalence of CBCL-defined cases in 11–14 years children than Frigerio et al. [53] in a 10–14 years urban community sample in ordinary setting (14.3% vs 9.8%). Our CBCL and YSR findings are consistent with PTSD results in young adults who received a clinical diagnosis according to DSM-IV nine months after the earthquake [32], while they are inconsistent with self-reported PTS symptoms in late adolescents and young adults in the same period [30,32]. Therefore, taking together these observations, we could argue that the investigation in a large community sample and the use of a culturally appropriate screening tool with satisfying psychometric properties as those we used, can help to obtain a more reliable estimation of psychiatric morbidity.

Age significantly affected the expression of psychiatric symptoms. Consistently with another other study based on diagnostic interview [24], pre-school children showed lower prevalence than elder children. Indeed, 6–10 years children had significantly higher burden associated with exposure to the earthquake, and their psychopathological pattern was consistent with most findings reported in literature [7,11,12,49]. In fact, a higher prevalence of CBCL-defined cases, anxiety and PTS symptoms was found in the epicentre at this age; also depressive symptoms were almost significantly higher in the epicentre. However, no association was found in our study between this age range and psychiatric outcomes. While on the one hand it may be inferred that the higher susceptibity may have been mediated by still immature coping strategies no longer mediated by the care-giver shell [3], on the other hand it is possible that non investigated factors, as in example a lack of social support, may have played relevant role for these children. Nevertheless, as children in this age group were not univocally considered vulnerable by studies in ordinary settings [53,54] and in complex emergencies [11,12,20,22,45-47], further investigation is needed to fully explain the observed developmental trend.

The expected small gender effect on psychiatric symptoms [11,55] was disconfirmed by our findings as also occurred in other studies [20,22,56]. As gender differences in internalizing symptoms typically emerge since puberty when these are more relevant in girls [57], it is possible that the relatively low prevalence of this symptoms spectrum in the whole sample and in the 11–14 years group may have reduced the effect of gender to non significant levels.

Our study extends the investigation from the well-established impact of exposure to disaster to health and family factors that may contribute to understand the role of ecological resources with regards to post-disaster outcomes [58].

Parents’ education had a protective role on children mental health, consistently with international literature [20,59] and extending previous association found in Italian pre-adolescents to earlier ages [53].

While the degree of exposure to the earthquake only affected PTS symptoms, psychiatric symptoms in children were strongly associated with different individual and family health conditions. Children’s chronic physical diseases doubled the risk of current psychiatric symptoms. Despite no other study examined this association in aftermath of disasters up to our knowledge, this finding supports the link between general and mental health often invoked in global mental health promotion [60]. It is to be wished that future research will take this major factor into account, because of the relevant implications for mental health services organization and integration with other health services in complex emergencies.

Moreover, the presence of premorbid conditions of mental sufferance in child or in his/ her mother showed the strongest and widest predictive value, raising this risk up to five – seven times. The impact of child pre-disaster and mother’s pre- and post-disaster mental health is consistent with results of several previous studies [19,20,30,56,59,61-67]. In our case, it may bolster two hypotheses: a direct trans-generational effect on child’s psychiatric outcomes, or a confounding effect on child’s symptoms she reports. This last instance may partly explain the disagreement between parent- and child-reported symptoms in the sense of an overestimation of CBCL symptoms prevalence. However, these findings underline the need of investigating prior conditions in research, and set a clear public health priority in planning post-disaster mental health and psychosocial interventions.

Strengths of the study are the large size and age range of the community sample, and the distribution of the enrolment sources consistent with the geographical distribution of all Regional FPs warranting to some extent the sample representativeness despite the convenience sampling procedure. Moreover, a wide range of disorders were examined by a psychometrically robust screening tool, and some pre-disaster factors were taken into account beyond trauma exposure.

The study also presents some limitations. Despite the above mentioned attempt to reduce biases our is a convenience sample, and the enrolling strategy may have contributed to a potential over-estimation of results. In fact, being the children enrolled in sequential order of presentation to consultation, it is possible that our sample consists of a higher proportion of children affected by physical illness or psychopathology, or cared by a disordered parent, than the general population. In addition, the self-report assessment may reduce reliability of some scales, where under- or over-estimations are generated by mothers or children themselves. Some predictive factors like other pre-disaster life events, pre- and post-disaster functioning, perceived threat and social support were not studied. Moreover, no baseline prevalence is available in Abruzzo children, thus limiting the possible inferences on the results.

## Conclusion

Twelve to 17 months after the L’Aquila earthquake, the number of children at risk of psychiatric disorders resulted relatively low and similar to the prevalence found in most western non emergency setting studies. Nevertheless, children aged 6–10 years living in the earthquake epicentre present a high risk of psychiatric disturbances, consistent with studies conducted in complex emergencies.

Our study confirms the need to empower the primary care system to improve the screening, monitoring, management and referral aimed at assessing and meeting the children’s mental health needs as part of the post-disaster interventions [68,69]. The study also highlights the importance of taking into account PTS, anxiety and depressive symptoms in complex emergencies, the importance of analysing predictive factors other than earthquake-related, and the relevance of adopting a multi-informant approach for both clinical implications and advance in research. Further epidemiological studies in non emergency settings would be very supportive for indicating a baseline that would warrant a deeper understanding of the effect of major events, such as disasters.

## Abbreviations

CBCL: Child Behavior Checklist
FPs: Family paediatricians
PTS: Post-traumatic stress
PTSD: Post-traumatic stress disorder
YSR: Youth Self Report
